# Application and performance of artificial intelligence-based models in the detection, segmentation and classification of periapical lesions: a systematic review

**DOI:** 10.3389/fdmed.2025.1717343

**Published:** 2025-11-24

**Authors:** Ali Alaqla, Sanjeev B. Khanagar, Alzahraa Ibrahim Albelaihi, Oinam Gokulchandra Singh, Abdulmohsen Alfadley

**Affiliations:** 1 Department of Restorative and Prosthetic Dental Sciences, College of Dentistry, King Saud bin Abdulaziz University for Health Sciences, Riyadh, Saudi Arabia; 2 King Abdullah International Medical Research Centre, Riyadh, Saudi Arabia; 3 Ministry of the National Guard Health Affairs, Riyadh, Saudi Arabia; 4 Department of Restorative Dentistry and Biomaterial Sciences, Harvard School of Dental Medicine, Boston, MA, United States; 5 Preventive Dental Science Department, College of Dentistry, King Saud Bin Abdulaziz University for Health Sciences, Riyadh, Saudi Arabia; 6 College of Dentistry, King Saud bin Abdulaziz University for Health Sciences, Riyadh, Saudi Arabia; 7 Radiological Sciences Program, College of Applied Medical Sciences, King Saud bin Abdulaziz University for Health Sciences, Riyadh, Saudi Arabia

**Keywords:** artificial intelligence, apical, conditions, classification, detection, neural networks, periapical, lesions and segmentation

## Abstract

**Background:**

Periapical lesions appear as periapical radiolucency on various imaging modalities. The accuracy of dentists in diagnosing periapical radiolucency varies significantly. Recent scientific and technological advancements have enabled the development and evaluation of artificial intelligence (AI) systems for various diagnostic applications in dentistry.

**Objectives:**

The aim was to report on the application and performance of AI-based models in the detection, segmentation, and classification of periapical lesions.

**Methods and methods:**

A systematic effort for data acquisition began with an exploration of a wide range of reputable databases, including PubMed, Scopus, Embase, Cochrane, Web of Science, Google Scholar, and the Saudi Digital Library. Our comprehensive investigation spanned from 1st January 2000 to 31st March 2025.

**Results:**

Twenty-eight articles fulfilled the eligibility criteria. Among these, 20 (71.4%) applied AI technology for automated detection, 3 (10.7%) for segmentation, 2 (7.2%) for periapical lesion detection and segmentation, and 3 (10.7%) for periapical lesion classification. Thirteen (46.5%) studies in this review utilized dental panoramic radiographs, 8 (28.5%) used intraoral radiographs (periapical and bitewing), and 7 (25%) employed CBCT scans. The AI models demonstrated an accuracy range of 70% to 99.65%, with sensitivity varying from 65% to 100% and specificity ranging from 62% to 100%. The risk of bias assessment using the QUADAS-2 tool, indicated 32.1% of the studies exhibited a significant risk of bias regarding the assessment of bias and applicability issues in the reference standard arm. While the certainty of evidence was evaluated through the GRADE approach, which indicated that the included studies demonstrated a moderate degree of evidence certainty.

**Conclusions:**

According to the results of the studies presented, AI-based technologies hold significant potential to assist clinicians and enhance the reliability of clinical diagnoses, enabling less experienced clinicians to identify lesions with greater accuracy. However, prospective studies and randomized clinical trials are essential to evaluate the effectiveness and cost-efficiency of deep learning-based lesion detection in real clinical settings.

**Systematic Review Registration:**

PROSPERO CRD420251011455.

## Introduction

Dental diseases continue to pose a significant public health challenge, affecting billions of individuals worldwide and resulting in substantial treatment costs (1). The WHO Global Oral Health Status Report (2022) estimates that oral disorders impact over 3.5 billion people globally, with three-quarters of those affected living in middle-income countries (2). The global prevalence of dental caries is particularly concerning, affecting nearly 2 billion individuals and increasing the risk of developing periapical lesions due to untreated or inadequately managed infections (3). Furthermore, periodontal diseases, which impact more than fifty percent of the global population, can facilitate the migration of bacteria into the periapical area, complicating both diagnosis and treatment (4).

Periapical lesions, commonly referred to as apical periodontitis, are characterized by localized inflammation of the periapical tissues resulting mainly from pulpal pathology. This condition may arise due to the progression of dental caries, trauma, or dental procedures. The host's immune response to harmful microorganisms initiates inflammation, which can ultimately lead to the destruction of periapical tissues (5). Apical diseases can exhibit a wide range of clinical manifestations, from the absence of noticeable signs or symptoms to significant deterioration of the underlying bone, with or without the presence of a draining abscess (6). A granuloma is the most common condition; however, it can progress into various other forms of disease, including an abscess, a periapical pocket cyst, or a true cyst, all of which manifest as radiolucencies (7).

Additionally, periapical radiolucencies can arise from extra-radicular infections, reactions to foreign bodies, and periapical scars, or they may result from other tumors and cysts that are not associated with pulp disease (8). It has been reported that approximately 52 percent of adults worldwide have encountered at least one tooth affected by periapical lesions (5), while the prevalence of granulomas ranges from 46% to 94%, and that of cysts from 6% to 55% (9). Certain lesions may develop pus, accompanied by pain and swelling, which could lead to systemic consequences if they are not identified or treated (10). It is essential for professionals to promptly identify, differentiate, and understand the various pathological entities and their dynamic interactions present in the periapical tissues to accurately diagnose and address these potentially dangerous situations before they deteriorate further (7, 11).

Pulp and periapical alterations can be assessed using various modalities, including cone beam computed tomography (CBCT), panoramic radiography, and intraoral periapical radiography. Among these, periapical radiographs obtained using the paralleling technique are the most effective for examining apical lesions, as they provide a comprehensive view of the tooth and its surrounding structures, including the periapical region and periodontium (12). However, these radiographs are always subject to inter- and intra-examiner variability, which can lead to diagnostic inconsistencies and potential errors (13). Recently, artificial intelligence (AI) has gained attention in the field of radiology for its potential as a diagnostic tool. Advances in deep learning, particularly in convolutional neural networks (CNNs), have facilitated the training and evaluation of AI systems for specialized diagnostic tasks.

AI has become an increasingly prominent trend in dentistry in recent years. It is currently utilized across various dental disciplines, including the detection of dental caries (14), identification of vertical root fractures (15), diagnosis of periodontitis, and categorization of potential forms of periodontal disease (16, 17). Additionally, AI aids in orthodontic treatment planning, outcome prediction, and assessing the need for extractions (18, 19). It is also employed for the detection of tumors and cancer through the analysis of radiographic, microscopic, and ultrasonographic images (20, 21). These research findings have demonstrated AI's capacity for learning and performance that surpasses human standards.

The ability of various neural networks to identify periapical lesions has been evaluated using a range of imaging methods, including periapical and panoramic radiographs as well as CBCT scans (22). Further investigation is necessary to determine the accuracy and effectiveness of utilizing AI in the diagnosis of periapical lesions through dentomaxillofacial radiographs, as this field is rapidly evolving. Therefore, the aim of this study was to systematically review and report on the application and performance of AI-based models for detecting, segmenting, and classifying periapical lesions.

## Materials and methods

To ensure the quality of the methods, this systematic review adhered to the diagnostic test accuracy requirements outlined by the Preferred Reporting Items for Systematic Reviews and Meta-Analyses Extension (PRISMA-DTA) (23) (Supplematary Table S1). The PICO (Population, Intervention, Comparison, Outcome) framework was applied to formulate the research question: What are the developments and performance of AI models used in the detection, segmentation, and classification of periapical lesions? The Population included patients who underwent investigations for periapical lesions using digital panoramic radiographs, periapical radiographs, CBCT images. The Intervention comprised AI applications designed for detection, segmentation, classification, and prediction of periapical lesions. The Comparison involved expert or specialist opinions, reference standards, or other AI models. The Outcomes considered measurable or predictive metrics such as [accuracy, sensitivity, specificity, recall, F1 score, precision, receiver operating characteristic (ROC) curve, area under the curve (AUC), area under the ROC curve (AUROC), intraclass correlation coefficient (ICC), intersection over union (IoU), statistical significance, mean average precision (mAP), positive predictive value (PPV), negative predictive value (NPV), Dice similarity coefficient (DSC), and mean absolute error (MAE)]. The protocol for this review was registered with PROSPERO with ID number CRD420251011455.

### Inclusion and exclusion criteria

The following criteria had to be met by the papers for inclusion: (a) they must be original studies focusing on AI technology; (b) they must provide measurable values that can be assessed or analyzed; and (c) they must discuss the data used to evaluate AI-based models. The study design for inclusion in this review was unrestricted. However, we excluded articles that did not address AI innovation, unpublished works or conference papers available online, case reports, editorial letters, review articles, opinion letters, articles lacking full-text versions, and those written in languages other than English.

### Search strategy

An electronic quest for data collection was initiated by exploring a wide array of reputable databases, including PubMed, Scopus, Embase, Cochrane, Web of Science, Google Scholar, and the Saudi Digital Library. Our persistent inquiry extended from the year 1st January 2000 to 31st March 2025. The following terms were utilized to search for articles: artificial intelligence, convolutional neural network, automated, machine learning, deep learning, supervised learning, unsupervised learning, x-rays, dental radiographs, panoramic radiographs, CBCT images, periapical, apical, conditions, lesions, abscess, radicular, cyst, granuloma, deep pulpal lesions, apical periodontitis, identification, detection, segmentation, classification and categorization. To locate articles within electronic databases, we employed Boolean operators (AND, OR) and applied filters for the English language.

(“artificial intelligence” OR “convolutional neural network” OR “automated” OR “machine learning” OR “deep learning” OR “supervised learning” OR “unsupervised learning” OR “x-rays” OR “dental radiographs” OR “panoramic radiographs” OR “CBCT images” OR “periapical” OR “apical” OR “conditions” OR “lesions” OR “abscess” OR “radicular” OR “cyst” OR “granuloma” OR “deep pulpal lesions” OR “apical periodontitis” OR “identification” OR “detection” OR “segmentation” OR “classification” OR “categorization”).

In addition to our electronic search, a thorough manual review of relevant research articles and their citations was conducted. This involved examining the reference lists of previously gathered articles in the college library, where physical copies of journals were available. The investigation was carried out by two independent individuals (A.A and S.B.K) who performed the task consistently.

### Study selection

The initial consideration included a total of 286 papers, of which 284 were identified through electronic database searches, while two were found manually. The selection process emphasized the relevance of the articles to the specific research domain, along with an assessment of the titles and abstracts. To ensure the elimination of duplicates, two individuals (A.F and A.I.A) not involved in the initial search meticulously reviewed all articles, resulting in the removal of 179 duplicates. In cases of disagreement during any screening stage, issues were resolved through consultation with a third reviewer (O.G.S). A thorough review of the remaining 105 full-text articles was conducted for data selection, applying the established eligibility criteria at this stage.

### Data extraction

Twenty-eight papers were selected for analysis after implementing the inclusion criteria. In the next phase, journal and author information was hidden to guarantee an objective assessment. Two authors (O.G.S and A.F) who were not involved in the initial search, were asked to evaluate the selected papers. The two reviewers demonstrated significant agreement, achieving an 88% level of concordance as assessed by Cohen's kappa.

**Figure 1:**
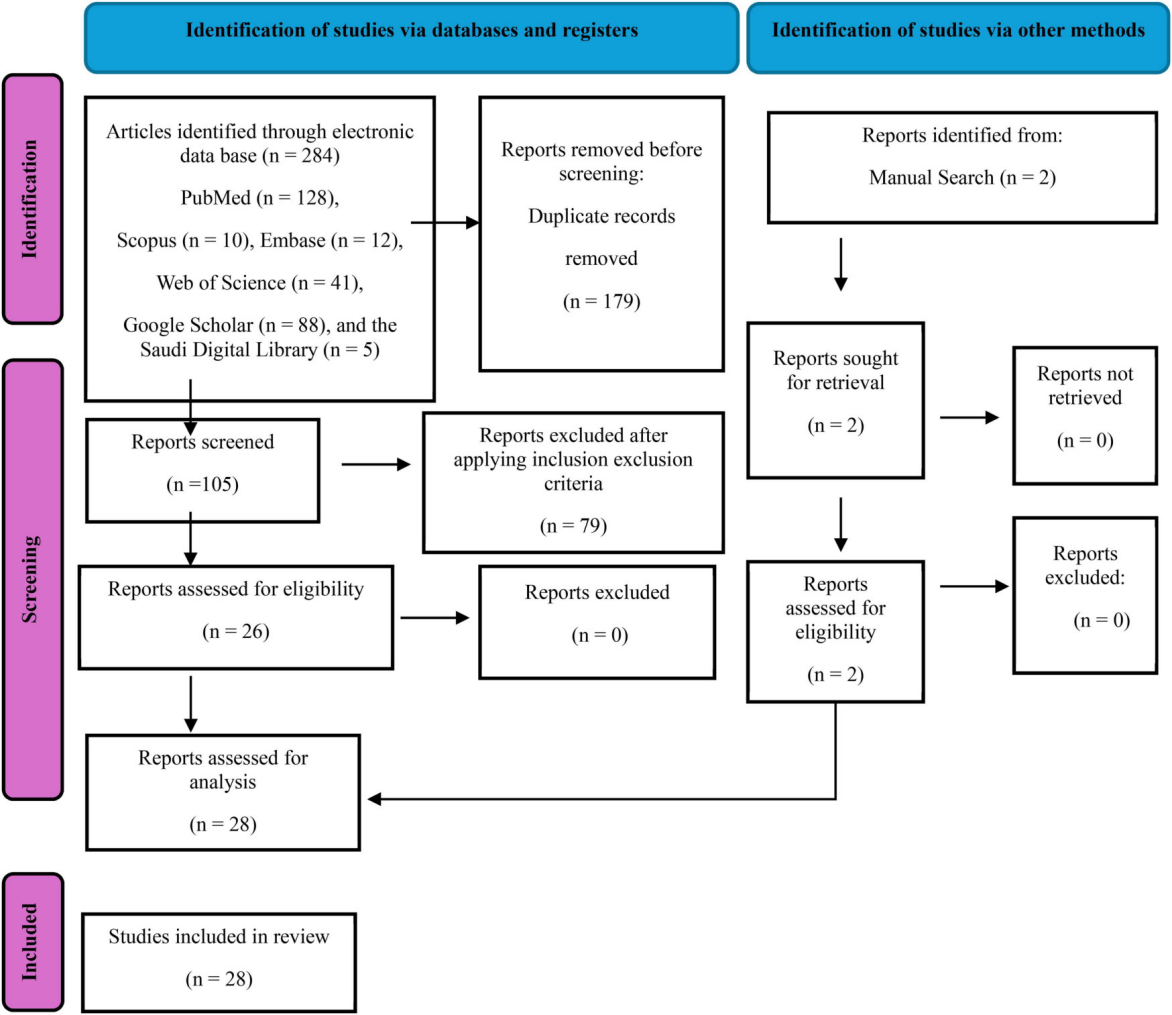
PRISMA 2020 flow diagram for new systematic reviews which included searches of databases, registers and other sources.

Information from the selected publications, including the authors' details, year of publication, study objectives, types of AI algorithms employed, and the data used for testing, validation, and training, as well as the results, conclusions, and recommendations, were extracted and entered in a Microsoft Excel document. Twenty-eight articles were identified as potentially eligible for qualitative synthesis in this systematic review and underwent critical analysis as illustrated in (Figure 1).

The included articles were evaluated for quality using QUADAS-2 (24), which assessed various aspects of study design and reporting, including patient selection, index tests, reference standards, and flow and timing. Each area is analyzed for potential risk of bias, with the first three areas also assessed for issues related to applicability. Signaling questions are included to aid in evaluating the risk of bias. This evaluation identified potential sources of bias and assessed the generalizability of the results across different clinical settings and patient populations.

## Results

A critical analysis of 28 articles was conducted, resulting in the extraction of qualitative data. Recent research from the past six years has shown a growing trend in the reporting of AI models used to detect periapical lesions.

### Qualitative synthesis of included studies

The primary applications of AI technology include automated detection (25–44), segmentation (45–47), periapical lesion detection and segmentation (48, 49), and periapical lesion classification (50–52). Thirteen of the 28 studies examined in this review utilized dental panoramic radiographs (25, 26, 29, 31, 33, 36, 37, 40, 43, 45–47, 52), eight utilized intraoral radiographs (periapical and bitewing) (30, 34, 35, 38, 41, 42, 44, 50), and seven employed CBCT scans (27, 28, 32, 39, 48, 49, 51) for training and testing as shown in (Table 1).

**Table 1:** Qualitative synthesis of included studies.

Sl No	Authors	Year of publication	Study Design	Algorithm Architecture	Objective of the study	No. of patients’/images/photographs for testing	Study factor	Modality	Comparison if any	Evaluation accuracy/average accuracy/ statistical significance	Results (+) effective (−) non effective (N) neutral	Outcomes	Authors suggestions/conclusions
1	Altukroni et al. (25)	2022	Cross-sectional	Multiple CNN algorithm	To design and evaluate an AI tool called Make Sure periapical explorer (MSp) to detect periapical lesions	2,200 DPRs (Allocated to training and data sets in the ratio of 8-1–1. 220)	Detection of periapical lesions	DPRs	Endodontists and general practitioners with more than two years of experience	MSp V/S Dentist Precision 0.88, 084 Recall 0.86,0.84 F1 Score 0.87,083	Effective	The model demonstrated higher performance in detecting periapical lesions when compared to the dentists group.	The model was reliable in detecting periapical lesions in DPRs
2	Ekert et al. (26)	2019	Cross-sectional	CNN	To detect apical lesions on DPRs.	2,001 tooth image segments	Detection of periapical lesions	DPRs	Independent and experienced dentists (clinical experience 3–10 years).	All teeth AUC = 0.85 ± 0.04 Sensitivity = 0.65± 0.12 Specificity = 0.87 ± 0.04 PPV = 0.49 ± 0.10 NPV = 93 ± 0.03.	Effective	The deep CNN model trained on a limited image data showed satisfying discriminatory ability in detecting apical lesions	This model can reduce dentists diagnostic efforts in detecting apical lesions. However, there is a need to further improve the sensitivity before clinical application.
3	Setzer et al. (27)	2020	Cross-sectional	DL (U net)	To automate detection of periapical lesions in CBCT images	20 CBCT images	Detection of periapical lesions	CBCT images	Clinician-dependent CBCT volume segmentation	DLS lesion detection Accuracy = 0.93 Specificity = 0.88 PPV = 0.87, NPV = 0.93.	Effective	This model demonstrated excellent accuracy in lesion detection.	With further enhancements of the model, the accuracy may be Benefited.
4	Hadzic et al. (28)	2023	Cross-sectional	CNN	To evaluate the performance and generalization capability of a deep learning-based periapical lesion detection algorithm	196 CBCT images	Detection of periapical lesions	CBCT images	Two senior oral surgeons with >15 years of experience and one junior dentist	Sensitivity = 86.7% Specificity = 84.3%.	Effective	The model can assist dentist in clinical practice.	Further improvements are needed to enhance the algorithm's robustness, particularly in detecting very small lesions and the handling of artifacts and outliers commonly encountered in real-world clinical scenarios
5	Chen et al. (29)	2021	Cross-sectional	R CNN	To train CNNs for lesion detections on DPRs and to evaluate performance across disease categories,	2,900 DPRs	Detection of periapical lesions	DPRs	Different AI networks	Periapical lesion IoU = 0.69 Precision = 0.516 Recall = 0.518	Effective	This research demonstrated that CNNs tend to identify lesions primarily at severe stages, and that training CNNs using a tailored strategy for each specific disease is more effective.	The CNNs can effectively apical lesions on radiographs
6	Shafi et al. (30)	2023	Cross-sectional	CNN (AlexNet)	To automate detection of periapical lesions	534 periapical images training and testing, containing 453 and 81 images,	Detection of periapical lesions	Periapical radiographs	Conventional classifiers (KNN classifier and the retrained (softmax classifier).	AlexNet Accuracy = 98% Precision = 0.757 Recall = 0.751 F1 Score = 0.748	Effective	The performance of the proposed method was evaluated against other conventional classifiers and achieved 98%.	The model can be used in everyday clinical practice to assist dentists in making decisions to enhance patient care.
7	Issa et al. (31)	2023	Cross-sectional	CNN	To evaluate the diagnostic accuracy of AI in detecting apical pathosis on DPRs	20 DPRs	Detection of periapical lesions	DPRs	One oral and maxillofacial radiology expert (more than ten years of experience) and one trainee in oral and maxillofacial radiology	Diagnocat Sensitivity = 92.3% Specificity = 97.8%. Accuracy = 96.66% F1 score = 0.92	Effective	The AI algorithm incorrectly identified one unhealthy tooth as healthy (false negative) and mistakenly labeled one healthy tooth as unhealthy (false positive) when compared to the actual results.	Diagnocat demonstrated excellent accuracy in identifying periapical periodontitis on periapical radiographs.
8	Kirnbauer et al. (32)	2022	Cross-sectional	CNN	To develop and validate a deep CNN for the automated detection of osteolytic PALs in CBCT data sets.	144 CBCT images	Detection of periapical lesions	CBCT images	Not mentioned	Success detection rate = 72.6%—97.3% Sensitivity = 97.1% Specificity = 88.0%	Effective	PALs exhibited differences in appearance, size, and shape within the CBCT dataset, and there was a significant imbalance between teeth with and without PALs; nevertheless, the proposed fully automated method delivered outstanding results.	The findings will offer an adequate foundation for future advancements, including those related to the incorporation of immature teeth.
9	Kumar et al. (33)	2023	Cross-sectional	CNN	To examine and compare the accuracy of several texture analysis techniques, such as Grey Level Run Length Matrix (GLRLM), Gray Level Co-occurrence Matrix (GLCM), and wavelet analysis in recognizing dental cyst, tumor, and abscess lesions.	172 DPRs	Detection of periapical lesions	DPRs	GLCM Wavelet analysis GLRLM	GLCM Accuracy = 98% Wavelet analysis Accuracy = 91.1% GLRLM Accuracy =95%.	Effective	The results obtained demonstrated excellent accuracy (98%) using GLCM, and these features can be utilized for future research.	Following enhancements in performance and training, it is capable of supporting routine histological diagnoses and aiding clinicians in developing accurate and timely treatment plans.
10	Li et al. (34)	2021	Cross-sectional	CNN	To identify the apical lesions of the periapical image through CNNs	476 tooth images (Validation-70% Testing-30%)	Detection of periapical lesions	Periapical radiographs	AlexNet, GoogleNet, Vgg, ResNet	Accuracy = 92.5% Sensitivity = 94.87% Specificity = 90.00% Precision = 92.50% Recall = 94.87%	Effective	The suggested model effectively enabled the automatic detection of the apical lesion.	This study paved the foundation for a new method to automate the detection of apical lesions on DPRs.
11	Hamdan et al. (35)	2022	Cross-sectional	CNN	To determine the efficacy of a DL tool in assisting dentists in detecting apical radiolucencies on DPRs	68 images	Detection of periapical lesions	Periapical radiographs	Eight Readers (Six operative Dentistry residents, a general dentist and an endodontist)	Average performance for all eight readers, the results of this study with the AI-aided session showed an 8.6% improvement in AFROC AUC Additionally, specificity by case and sensitivity by lesion showed 23.1 and 8.2% improvements, respectively	Effective	The study showed that the deep learning technology improves dental professionals’ capability to identify apical radiolucencies on intraoral radiographs.	This model has the potential to enhance dentists’ accuracy in detecting apical radiolucencies on periapical radiographs.
12	Boztuna et al. (36)	2024	Cross-sectional	CNN	To assess the diagnostic accuracy of AI model based on U²-Net architecture in the detection of periapical lesions on DPRs	400 DPRs	Detection of periapical lesions	DPRs	Not mentioned	Dice score = 0.8, Precision = 0.82 Recall = 0.77 F1-score = 0.8	Effective	The study demonstrated that an AI model utilizing the U²-Net architecture can precisely identify periapical lesions in panoramic radiographs.	The study shows that AI-based models have potential as helpful tools for dentists in identifying periapical radiolucencies and planning treatments.
13	Endres et al. (37)	2020	Cross-sectional	CNN	To assess the diagnostic accuracy of AI model in the detection of periapical lesions on DPRs	2,902 DPRs	Detection of periapical lesions	DPRs	24 oral and maxillofacial (OMF) surgeons	Average precision = 0.60 F1 score = 0.58	Effective	The study showed that the deep learning algorithm outperformed 14 out of 24 oral and maxillofacial surgeons.	This model, developed using a small dataset and tested against clinically validated reference standards, can aid OMF surgeons identify periapical lesions on panoramic radiographs.
14	Sajad et al. (38)	2019	Cross-sectional	CNN	To assess the diagnostic accuracy of AI in the detection of periapical lesions	534 periapical x-rays	Detection of periapical lesions	Periapical x-rays	Not mentioned	Accuracy SVM—98% Retrained—97% K-NN—95%	Effective	The SVM classifier trained on features extracted from the retrained model using data augmentation techniques demonstrated strong performance.	This model can assist the dentist to quickly identify the type of endoperio lesions in periapical radiographs.
15	Orhan et al. (39)	2019	Cross-sectional	CNN	To verify the diagnostic performance of CNN model To detect periapical pathosis on CBCT images.	153 periapical lesions	Detection of periapical lesions	CBCT images	Manual segmentation	Reliability = 92.8% Recall = 0.89 Precision = 0.95 F measure = 0.93	Effective	The AI system successfully detected 142 out of 153 periapical lesions. However, it mistakenly identified one tooth.	Volume measurements carried out by humans and AI systems were similar to one another.
16	Ünal et al. (40)	2024	Cross-sectional	CNN	To evaluate the function of diagnostic computer software designed for the detection of periapical lesions on panoramic images	500 panoramic images	Detection of periapical lesions	DPRs	Unclear	IoU = 0.8578 F1 score = 0.8587	Effective	The study showed that deep learning algorithms improve dental professionals’ ability to identify apical radiolucencies on intraoral radiographs.	Using AI to detect periapical lesions on panoramic radiographs will assist practitioners in making definitive diagnoses, even for lesions that might easily be missed.
17	Latke et al. (41)	2023	Cross-sectional	CNN	To detect periapical lesions using a hybrid model comprising of SVM and ANN	Dataset of 172 patients (80% to train 20% to test)	Detection of periapical lesions	Intra -oral Periapical radiographic images	KNN DT SVM ANN	Accuracy = 95.85% Precision = 0.97 Recall = 0.90 F1 score = 0.93	Effective	The model identified the anomalies with a 95.85% accuracy rate.	Further developments in the model further for refinement in endodontic treatments is suggested
18	Ngoc et al. (42)	2021	Cross-sectional	CNN	To assess the application of AI in support the diagnosis of periapical lesions.	1,30 bitewing images	Detection of periapical lesions	Bitewing images	Not mentioned	Sensitivity = 89.5% Specificity = 97.9% Accuracy = 95.6%,	Effective	The model can be utilized as an aid in diagnosing periapical lesions.	To assist in diagnosing periapical diseases in underserved areas with limited access to dentists
19	Güneç et al. (43)	2023	Cross-sectional	CNN	To evaluate the diagnostic performance based on radiological diagnoses regarding caries and periapical infection detection by comparing AI software with junior dentists	500 DPRs	Detection of periapical lesions	DPRs	Junior dentists who have 1 or 2 years of experience,	Sensitivity = 0.973, 0.962, 0.758, 0.958 Specificity = 0.629, 0.421, 0.404,0.621 PPV = 0.861, 0.651, 0.312,0.648 NPV = 0.689, 0.673, 0.278, 0.546 F1-score = 0.914, 0.777, 0.442, 0.773 for AI and three junior dentists respectively	Effective	AI software provided more precise results, particularly in identifying periapical lesions.	In terms of the time required for evaluation, AI was generally quicker.
20	Chuo et al. (44)	2022	Cross-sectional	CNN	To automate detection of peri apical lesions	490 periapical images Training-332 Validation-98	Detection of periapical lesions	Periapical radiographs	State-of-the-art ResNet101 ResNet 50 GoogleNet	Accuracy AlexNet = 96.21% ResNet101 = 94.70% ResNet 50 = 93.94% GoogleNet = 87.88%	Effective	The model effectively enhanced the automatic detection of apical lesions.	Automation allows dentists to concentrate more on technical and medical tasks, including treatment, tooth cleaning, and medical communication.
21	Ari et al. (45)	2022	Cross-sectional	CNN	To evaluate automatic segmentation performance of features in periapical radiographs by DL based AI model developed using U-Net algorithm.	1,169 DPRs	Segmentation of periapical lesions	DPRs	Not mentioned	Sensitivity = 0.92 Precision = 0.85 F1 Score = 0.88	Effective	This advanced AI model, based on the U-Net architecture, enhanced the accuracy of segmenting periapical lesions and root canal fillings on dental panoramic radiographs.	The findings of this study showed that deep learning systems using CNN architectures have the potential to help dentists segment various features in periapical images.
22	Bayrakdar et al. (46)	2022	Cross-sectional	D-CNN	To assess the diagnostic success of U-Net approach for the segmentation of apical lesions in DPRs	470 DPRs Training group: 380 Validation group: 43 Test group: 47	Segmentation of apical lesions	DPRs	Not mentioned	Sensitivity = 92% Precision = 84% F1-score = 88% IoU = 70%	Effective	AI systems have the capability to address clinical challenges. They can assist in evaluating periapical diseases using panoramic x-ray images.	Future research should involve larger sample sizes and images obtained from various radiography devices.
23	Song et al. (47)	2022	Cross-sectional	CNN	To evaluate the performance of a deep CNN algorithm for apical lesion segmentation from DPRs	1,000 DPRs (*n* = 800, validation (*n* = 100, and test (*n* = 100)	Segmentation of apical lesions	DPRs	Not mentioned	IoU = 0.3. F1 score = 0.828 Precision = 0.831 Recall = 0.826	Effective	In the test group consisting of 180 apical lesions, 147 lesions were successfully segmented, and the deep CNN algorithm based on U-Net showed significantly high accuracy in identifying apical lesions.	This study demonstrated the potential usefulness of a deep learning-based method for segmenting apical lesions.
24	Chau et al. (48)	2025	Cross-sectional	CNN	To investigate the efficacy of CBCT-SAM, a new AI model, in identifying periapical lesions on CBCT	659 CBCT images	Detection and segmentation of periapical lesions	CBCT images	Modified U-Net PAL-net	CBCT-SAM Detection Accuracy = 98.92% Average segmentation accuracy = 99.65% Sensitivity = 72.36%, Specificity = 99.87 Precision = 0.73 DSC = 0.70	Effective	CBCT-SAM is capable of offering expert-level support in detecting periapical lesions on CBCT scans.	In this study, CBCT-SAM demonstrated potential as an effective tool for detecting and segmenting lesions; however, additional research is needed to confirm its reliability and applicability across different cases.
25	Zheng et al. (49)	2021	Cross-sectional	CNN	To automate DL for CBCT multilabel segmentation and periapical lesion detection	Data sets of 20 patients	Detection and segmentation of periapical lesions	CBCT images	Standard Dense U-Net	Dense U net (multiclass logistic loss plus dice loss) Accuracy = 0.81 Recall = 0.78	Effective	Our experiment showed that the proposed algorithm surpasses the standard Dense U-Net in terms of lesion detection accuracy and dice coefficient metrics for multilabel segmentation.	Taking advantage of the integration with anatomical domain knowledge, this algorithm achieves good performance even when trained on data from a limited number of patients.
26	Fatima et al. (50)	2023	Cross-sectional	Mask RCNN	To propose an automated model for periapical disease detection	534 periapical images (80% for training, 10% for validation, and the remaining 10% for testing)	Classification and localization of different periodontal lesions	Periapical radiographs	Base RCNN using the ResNet-50 as a backbone network	Accuracy = 94%, Ma*p* = 85%, mIOU = 71.0%.	Effective	The proposed model significantly enhanced detection, classification, and localization accuracy while using fewer images than existing methods, outperforming state-of-the-art techniques.	Furthermore, the model's performance can be enhanced by using a larger annotated dataset containing multiple lesions.
27	Calazans et al. (51)	2022	Cross-sectional	CNN	To automate classification system for periapical lesions	1,000 sagittal and coronal sections of cone-beam CT scans	Classification of periapical lesions	CBCT images	DenseNet Vs VGG-16 0.	DenseNet-121 Accuracy = 70% F1-Score = 69.7% Specificity = 76.34% Precision = 75.82% Recall = 64.49% VGG-16 Accuracy = 68% F1-Score = 68.32% Specificity = 72.04% Precision = 72.63% Recall = 64.49%	Effective	An accuracy of approximately 70% was achieved for the entire image set, 81.25% for the images without lesions and those with large lesions, and 66.67% for the images without lesions and those with small lesions.	The results highlight the superiority of DenseNet-121, as it outperformed in four of the five evaluated metrics.
28	Ver Berne et al. (52)	2023	Cross-sectional	CNN	To develop a DL approach for classification and localization of radicular cysts and periapical granulomas of the mandible on panoramic imaging	249 panoramic images	Classification of radicular and periapical cyst	DPRs	Two dento-maxillofacial radiology experts	MobileNetV2 Radicular cysts Sensitivity = 100% Specificity = 95% AUC = 97% Precision = 83% Periapical granuloma Sensitivity = 77 Specificity =100 AUC = 88% Precision = 74%	Effective	The suggested model showed consistent accuracy in diagnosing and distinguishing between radicular cysts and periapical granulomas.	Utilizing deep learning can improve diagnostic accuracy, resulting in a more effective referral process and better treatment outcomes.

MSp, make sure periapical explorer; ML, machine learning; DL, deep learning; ANNs, artificial neural networks; CNNs, convolutional neural networks; DCNNs, deep neural networks; MLP, multi-layer perception; DT, decision tree; SVM, support vector machine; C-index, concordance index; CT- scans, computed tomography; CBCT, cone-beam computed tomography; OCT, optical coherence tomography; NBC, naïve bayes classifier; WE, wavelet energy; LR, logistic regression; SVM, support vector machine; CLAHE + GLCM + ELM, contrast limited adaptive histogram equalization, gray level co-occurrence matrix, and extreme learning machine; DSC, dice similarity coefficient; DPR, dental panoramic radiographs; Ag P, aggressive periodontitis; CP, chronic periodontitis; LM, levenberg—marquardet; SCG, scaled conjugate gradient; RBL, radiographic alveolar bone level; ABL, alveolar bone loss; MGLCM, multichannel gray—level co—occurrence matrix; PSONN, particle swarm optimization neural network; GLRLM, grey level run length matrix; GLCM, grey level co-occurrence matrix; AFROC, alternative free-response receiver operating characteristic.

### Characteristics of studies

All 28 articles included in the review have employed a cross-sectional study design and were published between 2019 and 2025. The models developed were mostly based on CNNs architectures.

### Outcome measures

The evaluation of task performance efficiency was conducted using various outcome measures, which included quantifiable and predictive results such as accuracy, sensitivity, specificity, recall, precision, F1 score, receiver operating characteristic curve (ROC), area under the curve (AUC), area under the receiver operating characteristic (AUROC), intraclass correlation coefficient (ICC), intersection over union (IoU), statistical significance, mean average precision (mAP), positive predictive value (PPV), negative predictive value (NPV), dice similarity coefficient (DSC), and mean absolute error (MAE).

Overall, the AI models demonstrated promising results and enhanced dental professionals' ability to detect apical lesions. These models can play a significant role in assisting dentists in clinical practice. AI systems have the potential to effectively address clinical challenges. One study showed that the AI model achieved higher performance metrics in detecting periapical lesions compared to a group of dentists (25). Another study demonstrated that the deep learning (DL) algorithm outperformed 14 of 24 oral and maxillofacial (OMF) surgeons (37). Additionally, a further study reported that the AI model can provide expert-level assistance in identifying periapical lesions on CBCT scans (48). However, a moderately deep convolutional neural network (DCNN) trained on a limited amount of image data showed satisfactory discriminatory ability to detect lesions (26). Another study reported that the AI algorithm misdiagnosed one unhealthy tooth (false negative) and overdiagnosed one healthy tooth (false positive) compared to the ground-truth results (31).

### Evaluation of bias risk and considerations of applicability

The QUADAS-2 tool, was utilized to evaluate the quality and assess the risk of bias associated with the included studies. This tool is a widely used and validated instrument for evaluating the methodological quality of primary diagnostic accuracy studies included in systematic reviews. It assesses studies across four essential domains—patient selection, the index test, the reference standard, and flow and timing—by examining each domain for potential bias and concerns regarding its applicability to the review question (24). All studies incorporated patient-derived datasets (data used to train, validate, and test the AI models) including dental radiographs, cone beam CBCT scans, and photographic images, as inputs for CNNs and artificial neural networks (ANNs), thereby minimizing the potential for bias in the patient selection domain across both arms. The index test domain exhibited a minimal risk of bias in both arms of QUADAS-2, attributable to the implementation of a consistent training methodology throughout all investigations. Furthermore, the bias associated with the flow and timing domain was further mitigated through the application of standardized methodologies for data input in AI technology. However, the reference standard for interpreting the findings of the index test was not clearly defined in seven of the included studies, raising concerns about potential biases in the index test, reference standard, flow, and timing domains within both the risk of bias and applicability components.

**Figure 2:**
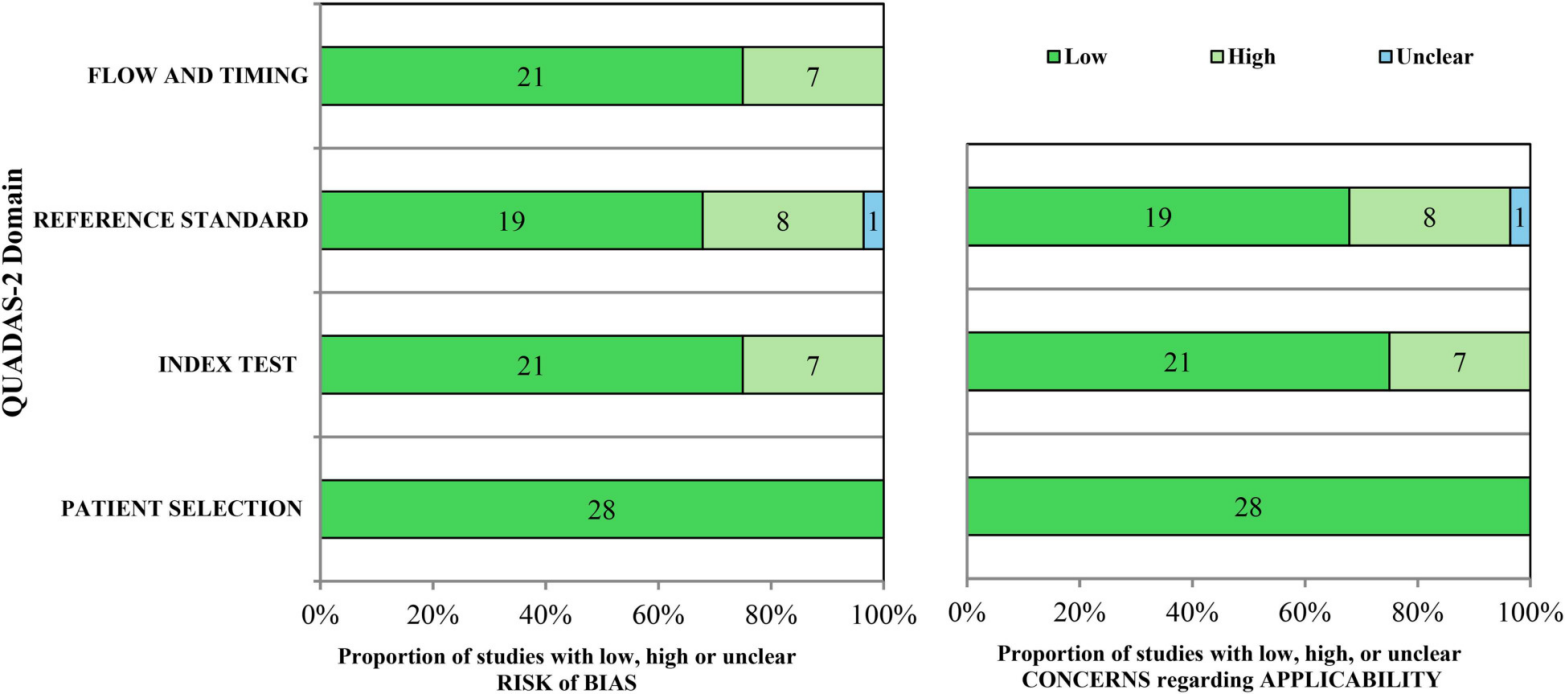
QUADAS-2 assessment of the individual risk of bias domains and applicability concerns.

One of the included studies utilized observations from less experienced dentists as a reference standard (43), while the reference standard for another study is unclear (40). Consequently, 32.1% of the studies exhibited a significant risk of bias regarding the assessment of bias and applicability issues in the reference standard arm. An analysis of all categories in the included trials indicated a low probability of bias in both groups. The evaluation of bias risk and applicability issues for the included research is detailed in the (Figure 2 and Supplementary Table S2).

This systematic review utilized the Grading of Recommendations Assessment, Development, and Evaluation (GRADE) methodology to evaluate the level of certainty in the evidence (53). The confidence in the evidence is classified as extremely low, low, moderate, or high based on five factors: publication bias, indirectness, imprecision, risk of bias, and inconsistency. This assessment indicated that the studies included in this systematic review demonstrated a moderate degree of evidence certainty (Table 2).

**Table 2:** Assessment of strength of evidence.

Outcome	Inconsistency	Indirectness	Imprecision	Risk of bias	Publication bias	Strength of evidence
Application of AI to detect periapical lesions (25–44)	Not Present	Not Present	Not Present	Present	Not Present	⨁⨁⨁○
Application of AI in segmentation of periapical lesions (45–47)	Not Present	Not Present	Not Present	Present	Not Present	⨁⨁⨁○
Application of AI in detection and segmentation of periapical lesions (48, 49)	Not Present	Not Present	Not Present	Not Present	Not Present	⨁⨁⨁⨁
Application of AI in classification of periapical lesions (50–52)	Not Present	Not Present	Not Present	Not Present	Not Present	⨁⨁⨁⨁

⨁⨁⨁⨁—high evidence ⨁⨁⨁○—moderate evidence.

### Overall summary of the included studies

Among the 28 included articles, 20 (71.4%) applied AI technology for automated detection, 3 (10.7%) for segmentation, 2 (7.2%) for periapical lesion detection and segmentation, and 3 (10.7%) for periapical lesion classification. Thirteen (46.5%) studies in this review utilized dental panoramic radiographs, 8 (28.5%) used intraoral radiographs (periapical and bitewing), and 7 (25%) employed CBCT scans. The AI models demonstrated an accuracy range of 70% to 99.65%, with sensitivity varying from 65% to 100% and specificity ranging from 62% to 100%. The risk of bias assessment using the QUADAS-2 tool indicated that 32.1% of the studies exhibited a significant risk of bias concerning the assessment of bias and applicability issues in the reference standard domain. The certainty of evidence was evaluated using the GRADE approach, which indicated that the included studies demonstrated a moderate level of evidence certainty.

## Discussion

An accurate diagnosis requires a comprehensive clinical and radiographic evaluation, along with meticulous documentation of the patient's medical and dental history. In some cases, a diagnosis may remain ambiguous despite exhaustive investigations (54). Dental radiographs are essential diagnostic tools that aid in visualizing oral diseases, developing treatment strategies, and monitoring patient progress (55). Instances of incorrect or inadequate diagnoses have been reported, even though dentists invest significant time in interpreting radiographs. This can result from subjective factors such as fatigue, emotions, and inexperience, which can impair judgment (56). The potential for bias due to inter- or intra-examiner reliability cannot be overlooked. Additionally, the complex anatomy and the ever-changing nature of diseases make it challenging for dentists to quickly and accurately interpret radiographs (56). The current methods of image evaluation and annotation require dental professionals to perform these tasks manually, which increases their workload (56). The efforts of dentists can be alleviated, and the likelihood of misdiagnosis can be reduced by utilizing automated systems for radiographic interpretation (57). In this way, AI models have enhanced the accuracy and efficiency of dental disease detection and prognosis (58), helping to achieve precise interpretations and minimizing human error (59). In the present systematic review, we witnessed that there was a wide variation in the datasets used for training the models. 43% of the included studies used panoramic radiographs and 28.5% studies have used periapical radiographs and CBCT images respectively which has resulted in the heterogeneity of the datasets and making it difficult to perform meta-analysis at this stage. It is important to note here that 2D radiographs allow limited accuracy in comparison with the 3D imaging for detecting the periapical lesion (32, 52). We also observed that few studies employed only one dentist are reference standard and around 25% of the studies do not clearly mention on the reference standards. Comparing the model's performance with only few clinicians' interpretations is prone to human errors, which is a common issue across the studies reporting on the performance of the models (60). This systematic review explores application and performance of AI in the segmentation, classification of periapical lesions.

### Application of AI models in detection of periapical lesions

In the pool of articles investigated in our research, 20 have examined the application of AI in the detection of periapical lesions. One of the most remarkable studies was conducted by Shafi et al. (30), who designed an automated diagnostic system utilizing Internet of Things (IoT) technologies and deep learning strategies for dental lesion diagnosis, eliminating the need for manual intervention from either the patient or the physician. The system employed data augmentation and transfer learning to enhance the performance of a pre-trained AlexNet CNN. Classification was performed using support vector machines (SVM) and k-nearest neighbors (KNN), along with a retrained network featuring a Softmax classifier. The proposed model demonstrated varying degrees of accuracy in identifying dental lesions, achieving the highest accuracy of 98% when using the SVM classifier. While the study highlighted the effectiveness of the proposed model, it did not conduct direct empirical benchmarking to estimate its relative accuracy compared to other models (30).

In another study, Kumar et al. (33) employed a range of feature extraction methods, including the Gray Level Co-occurrence Matrix (GLCM), Gray Level Run Length Matrix (GLRLM), and wavelet analysis, to extract data from the area of interest. The results indicate that an accuracy of 98% was achieved using GLCM, 91% accuracy was obtained via wavelet analysis, and 95% accuracy was reached with GLRLM in differentiating among dental cysts, tumors, and abscess lesions. The area under the curve (AUC) values suggest that GLCM provides a significant level of accuracy. GLCM can assist clinicians in formulating precise and timely treatment strategies. However, it is important to note that models trained and tested on identical datasets, and not subjected to an independent data, raises concerns about their validation (33).

Sajad et al. (38) designed a model that could automatically identify lesions on periapical radiographs using a two-channel training structure with the pre-trained AlexNet model for feature extraction. Then, these features were categorized using SVM and KNN classifiers achieving an accuracy of 98% from SVM classifier. However, this study did not specify about the types of lesions detected, which limits of applicability of its findings in broader diagnostic instances. Additionally, the use of limited datasets raises questions about generalizability of its findings (38).

Latke et al. (41) proposed a hybrid model for the detection of periapical lesions, focusing on three major aspects: image processing, machine learning and deep learning methodologies. The overall performance of the AI model's model 95.85%, which was realtively low accuracy in comparison to traditional machine learning models, such as KNN (100%), Decision Tree (DT) (100%), SVM (99.61%), and ANN (99.56%). In contrast, the higher accuracy levels reported using KNN and DT may be due to their effectiveness in dealing with small datasets. Even though the hybrid model was not observed to be the most accurate, this seemed to proved very useful in integrating AI techniques into real world (41).

Endres et al. (37) conducted performance evaluations of oral and maxillofacial (OMF) surgeons alongside an AI-based algorithm to assess their ability to identify periapical radiolucencies. OMF surgeons demonstrated a mean positive predictive value (PPV) of 0.69, which corresponded to a false-positive rate of 31%, and achieved a mean true positive rate (TPR) of 0.51, indicating a failure to detect 49% of actual periapical radiolucencies. The diagnostic performance metrics illustrated that even skilled professionals encounter challenges when attempting to detect periapical diseases from panoramic radiographs. The deep learning algorithm produced average precision and F1 scores of 0.60 and 0.58, respectively. The PPV measurement was 0.67, while its TPR matched the surgeons' results at 0.51. The AI system achieved performance levels comparable to human experts but demonstrated slight improvements in specific areas of detection accuracy. The research also revealed that the deep learning algorithm outperformed 14 out of the 24 OMF surgeons who participated in the study, suggested that AI could become an essential assistive tool for detecting periapical diseases (37).

Chuo et al. (44) proposed a CNN-based regional analysis approach to enhance the diagnosis of apical lesions in periapical radiographs through transfer learning, achieving a detection accuracy exceeding 96%. This was accomplished by employing an adaptive threshold pre-processing technique to improve segmentation accuracy. Additionally, the model's capability to differentiate pathogenic zones was also enhanced the development of a symptom amplification technique, which increased the appearance of lesion areas. However, there was no direct comparison of the results to other deep learning models or traditional image processing methods which can be considered as a drawback (44).

### Application of AI models in segmentation of periapical lesions

Automatic feature segmentation in dental periapical radiographs has demonstrated significant improvements in accuracy, speed, and diagnostic reliability when deep learning techniques-specifically CNNs and U-Net architectures-are applied. To automatically differentiate various dental features in periapical radiographs, Ari T et al. (45) developed a U-Net model that demonstrated excellent accuracy in segmenting periapical lesions while balancing the trade-off between false positives and false negatives, achieving sensitivity, precision, and F1-scores of 0.92, 0.85, and 0.88, respectively. Additionally, in early-stage lesions where radiolucency is subtle, AI models have struggled to distinguish between normal periapical structures and malignant alterations. Furthermore, the lack of contrast in typical periapical radiographs may lead to classification of lesions that overlap with root canal fillings, restorations, or thick surrounding bone structures to be misclassified (45).

The efficiency of a U-Net-D-CNN for apical lesion segmentation in panoramic dental radiographs was investigated by Bayrakdar et al. (46). The U-Net model demonstrated impressive results, achieving a sensitivity of 0.92, precision of 0.84, and an F1-score of 0.88 at a 70% intersection over union (IoU) threshold, highlighting its ability to accurately delineate periapical lesions. Despite these promising outcomes, the study did not include direct comparisons with other deep learning models or segmentation networks, which would have provided additional evidence regarding its advantages or limitations in this task (46).

### Application of AI models in detection and segmentation of periapical lesions

Chau et al. (48) developed an AI model known as CBCT-SAM, which detects periapical lesions in cone beam computed tomography (CBCT) scans. The AI model was trained and validated using 185 CBCT scans that contained diagnosed periapical lesions, achieving an average diagnostic accuracy of 98.92% and a segmentation accuracy of 99.65%. In terms of segmentation accuracy, CBCT-SAM significantly outperformed the Modified U-Net model. The inclusion of the progressive prediction refinement (PPR) module resulted in a slight enhancement in both diagnostic and segmentation tasks. The efficiency of clinical workflows improved with CBCT-SAM, as it reduced diagnostic time by 40%. These results indicate CBCT-SAM can be a expert-level support in detecting periapical lesions across CBCT scans. However, it has also been observed that variations in CBCT image quality, scanner settings and bone density of patients adversely affect the performance of these models. Additionally, in general, this model tends to detect more aggressive lesions and was slightly less accurate with respect to small or peculiar lesions, particularly in scenarios with overlapping organs (48).

To enhance segmentation and lesion detection, Zheng et al. (49) proposed a technique that integrates anatomical constraints into the Dense U-Net architecture. By utilizing anatomical data, the system aligns more closely with actual dental anatomy, thereby reducing errors commonly associated with traditional deep learning models. This approach demonstrated improved performance compared to standard Dense U-Net models, particularly in multi-label segmentation tasks. It achieved a lesion detection accuracy of 84% and improved Dice Coefficient (DICE) indices. Furthermore, this research indicated that the deep learning framework maintained significant efficacy even when trained on a very small dataset, suggesting that the method is effective in identifying critical patterns without the need for extensive labeled data which is a common limitation in medical imaging research. This anatomically constrained deep learning approach reduces the need for manual annotations and enhances diagnostic accuracy, potentially optimizing CBCT analysis and assisting dentists and radiologists in achieving faster and more reliable diagnoses (49).

### Application of AI models in classification of periapical lesions

Radicular cysts and periapical granulomas are two common lesions identified in the jaw through panoramic imaging (60). Differentiating between these lesions is crucial, as it significantly impacts treatment decisions. Radicular cysts can persist and even proliferate independently of their original inflammatory stimulus, while granulomas are typically managed with a root canal procedure, which eliminates the inflammatory stimulus within the root canals. Distinguishing between periapical granulomas and radicular cysts using conventional radiographic techniques is a time-consuming and complex task, even for experienced professionals (61, 62).

Ver Berne et al. (52) investigated the efficacy of an AI model in differentiating radicular cysts from periapical granulomas using panoramic imaging. This model achieved a sensitivity of 100% and a specificity of 95% for radicular cysts. The sensitivity and specificity for periapical granulomas were 100% and 77%, respectively. The localization network's average accuracy was 83% for radicular cysts and 74% for periapical granulomas. When compared to general dentists, the results demonstrated that the deep learning model was highly effective in avoiding misdiagnosis of periapical granulomas. However, the reduced sensitivity for granulomas (77%) indicates the need for further improvements to the model, as it suggests that some actual cases may have gone undetected (52).

Fatima et al. (50) utilized an enhanced MobileNet-v2 backbone to develop a streamlined version of Mask R-CNN for the classification and localization of five distinct periapical lesions. The model achieved an overall accuracy of 94% and an average precision of 85%. Despite being trained on a small dataset, the model outperformed baseline classification and localization methods (50).

### Limitations and future considerations in AI

In this review, studies examining the performance of AI models designed for the detection of periapical lesions have shown promising results. It is widely acknowledged that AI systems have the potential to transform and revolutionize dental practice, particularly in performing repetitive tasks and analyzing vast volumes of data (63). However, several other factors must be considered for the successful implementation of AI in clinical settings.

The effective development of an automated system relies heavily on the availability of large and diverse datasets, as they enable models to learn complex patterns and improve accuracy. Data augmentation techniques, which artificially expand datasets through various transformations, help to address the challenge of limited data (64). Additionally, it is essential that the training datasets have low error rates and are reliable and consistent to ensure robustness. Unfortunately, several studies included in this review utilized relatively small datasets, which may have limited their ability to generalize effectively (65). This limitation arises because AI algorithms often require substantial amounts of data to extrapolate accurately to a variety of scenarios. Therefore, the sample size must be justified and, if feasible, statistically calculated to mitigate this issue (66).

Moreover, developing accurate and effective tools for annotation, labeling, and drawing is essential for successfully executing AI tasks. The studies included have utilized panoramic radiographs, periapical radiographs, and CBCT images. In the present systematic review, we witnessed that there was a wide variation in the datasets used for training the models. 43% of the included studies used panoramic radiographs and 28.5% studies have used periapical radiographs and CBCT images respectively which has resulted in the heterogeneity of the datasets. It is important to note here that 2D radiographs allow limited accuracy in comparison with the 3D imaging for detecting the periapical lesion (32, 52). We also observed that few studies employed only one dentist are reference standard and around 25% of the studies do not clearly mention on the reference standards. Comparing the model's performance with only few clinicians' interpretations is prone to human errors, which is a common issue across the studies reporting on the performance of the models (60). Documenting the number of human annotators involved and their qualifications is vital for ensuring the quality of the labeled data (67). Steps should be taken to minimize inter- and intra-rater variability, thereby enhancing the reliability of the reference standard. Approximately one-quarter of the studies included in this research did not have a reference standard, which complicates the process of conducting relative comparisons and increases the risk of bias (68).

All studies included in this systematic review are retrospective and rely on pre-existing datasets. Although AI-based diagnostic models demonstrate high accuracy and sensitivity, they are not yet sufficiently reliable for independent use in real clinical settings. Prospective studies and randomized clinical trials are essential to evaluate the effectiveness and cost-efficiency of deep learning-based lesion detection in real clinical settings. Difficulty in interpreting the reasoning behind inaccurate judgments poses a significant challenge in identifying and rectifying the foundational logic employed by AI. The issue of accountability in the event of errors raises complex ethical dilemmas for clinicians using AI tools, as the distinction of responsibility may become ambiguous. In a clinical setting, integrating AI technologies necessitates substantial investment in infrastructure and extensive training for dental professionals. This can be particularly challenging for smaller practices or those operating with limited resources. Furthermore, safeguarding patient privacy and ensuring data security represent critical hurdles, as AI relies on sensitive health information. It is essential to adhere strictly to regulations that protect privacy and comply with legal standards.

## Conclusions

According to the results of the studies presented, AI-based technologies hold significant potential to assist clinicians and enhance the accuracy of clinical diagnoses. The AI models demonstrated an accuracy range of 70% to 99.65%, with sensitivity varying from 65% to 100% and specificity ranging from 62% to 100%. The articles underwent risk assessment using the QUADAS-2 tool, and indicated 32.1% of the studies exhibited a significant risk of bias regarding the assessment of bias and applicability issues in the reference standard arm. While the certainty of evidence was evaluated through the GRADE approach, which indicated that the studies included in this systematic review demonstrated a moderate degree of evidence certainty. This comprehensive review revealed that AI models outperformed other models in diagnosing, segmenting, and classifying periapical lesions in terms of accuracy, sensitivity, specificity, precision, and recall. Prospective studies and randomized clinical trials are essential to evaluate the effectiveness and cost-efficiency of deep learning-based lesion detection in real clinical settings.

## Data Availability

The original contributions presented in the study are included in the article/Supplementary Material, further inquiries can be directed to the corresponding author.
